# *Cryptosporidium* infections in suckler herd beef calves

**DOI:** 10.1017/S0031182015000426

**Published:** 2015-04-22

**Authors:** C. BJÖRKMAN, L. LINDSTRÖM, C. OWESON, H. AHOLA, K. TROELL, C. AXÉN

**Affiliations:** 1 Department of Clinical Sciences, Swedish University of Agricultural Sciences, P.O. Box 7054, SE-750 07 Uppsala, Sweden; 2 Department of Virology, Immunobiology and Parasitology, National Veterinary Institute, SE-751 89 Uppsala, Sweden; 3 Department of Animal Health and Antimicrobial Strategies, National Veterinary Institute, SE-751 89 Uppsala, Sweden

**Keywords:** *Cryptosporidium*, beef calves, suckler herd, diarrhea, *Cryptosporidium bovis*, *Cryptosporidium parvum*, *Cryptosporidium ryanae*, *Cryptosporidium ubiquitum*

## Abstract

A study was carried out to investigate how common *Cryptosporidium* infections are in beef calves in Swedish suckler herds and to explore which species and subtypes that occur. We further aimed at identifying factors associated with shedding of *Cryptosporidium* oocysts in this type of calf management. The study was conducted in two regions in Sweden and included 30 herds. Faecal samples were collected from calves younger than 3 months. A brief clinical examination was done and a questionnaire was used to collect data on management routines. Faeces were cleaned and concentrated and oocysts identified by epifuorescence microscopy. *Cryptosporidium* positive samples were analyzed at the 18S rRNA and GP60 genes to determine species and *Cryptosporidium parvum* subtype, respectively. Logistic regression was used to identify factors associated with infection. Oocysts were detected in 122 (36·7%) calves from 29 (97%) herds, at 400 to 2·4 × 10^7^ OPG. The youngest positive calves were only 1 and 2 days old. There was no association between age and *Cryptosporidium* infection. *Cryptosporidium bovis, Cryptosporidium ryanae, C. parvum* and *Cryptosporidium ubiquitum* were identified, with *C. bovis* being the major species. Two *C. parvum* subtypes, IIaA16G1R1 and IIdA27G1 were identified. Routines for cleaning calf pens and number of cows in calving pens were associated with infection.

## INTRODUCTION

The protozoan genus *Cryptosporidium* are clinically important pathogens causing gastrointestinal disease in a variety of species, including cattle and humans (Cacciò and Widmer, 2014). The parasites are transmitted as oocysts via the faecal-oral route, either by individual-to-individual contact, by exposure to contaminated equipment or by ingestion of contaminated food or water. *Cryptosporidium parvum* is one of the most common causes of calf diarrhoea worldwide (Blanchard, 2012). One infected calf can shed billions of oocysts in the faeces, and thus effectively spread the infection within the herd and to the environment. Cattle are mainly infected with four *Cryptosporidium* species, of which three are morphologically identical but genetically different species; *C. parvum, C. bovis* and *C. ryanae*; whereas *C. andersoni* can be distinguished by its larger, ovoid oocysts. *C. parvum* has a broad host range and infects most mammals including humans and cattle whereas *C. bovis* and *C. ryanae* seem to be adapted to cattle (Robertson *et al.* 2014).

Bovine *Cryptosporidium* spp. infections are common around the world and infections are most common in preweaned calves. In studies comprising dairy calves up to 2 months of age, 5–93% of investigated calves shed oocysts and the cumulative prevalence was 100% in some herds (O'Handley *et al.* 1999; Santín *et al.* 2008). The overall picture is that there is an age related pattern in the distribution of *Cryptosporidium* species. *C. parvum* is mostly found in young calves up to 2 months of age, whereas *C. bovis* and *C. ryanae* are the most common species in older calves and young stock (Robertson *et al.* 2014). *C. andersoni* is mainly found in young stock and adult cattle (Robertson *et al.* 2014).

*Cryptosporidium* species have been shown to be ubiquitous in Swedish dairy herds; with the highest infection rates in calves, followed by young stock and cows (Silverlås *et al*. 2009; Silverlås & Blanco-Penedo, 2013). In the studies by Silverlås *et al.* (2009); and Silverlås & Blanco-Penedo (2013) *C. parvum* was only found in calves up to 9 weeks of age, although *C. bovis* was the dominating species even in this age group. This is in contrast to what has been reported from many other countries (Langkjaer *et al.* 2007; Plutzer & Karanis, 2007; Santín *et al.* 2008; Brook *et al.* 2009). Analysis of the GP60 locus can be used to discriminate between species specific and zoonotic *C. parvum* subtypes. All Swedish bovine isolates analysed so far belong to the zoonotic subtype families IIa and IId (Silverlås *et al.* 2010*a*, 2010*b*, 2013; Silverlås and Blanco-Penedo, 2013).

In the 1970s the number of herds with specialized beef breeds started to increase in Sweden and today approximately a third of all Swedish cows are kept in cow-calf production systems. In 2012, there were 11 375 suckler herds with an average of 17 cows per herd (Statistics Sweden, 2013). In contrast to calves in dairy herds, calves in suckler herds are kept on pasture with their dams. In recent years questions concerning the risk of spread of zoonotic pathogens like *C. parvum* from grazing animals to water have been frequently discussed in Sweden. In order to assess the extent of *C. parvum* shedding into the environment from suckler herds and the potential zoonotic implications thereof, this study was implemented. The main objectives were to investigate how common *Cryptosporidium* infection is in calves in Swedish suckler herds and to explore which species and subtypes that occur. We further aimed at identifying factors associated with shedding of *Cryptosporidium* oocysts in this type of calf management.

## MATERIALS AND METHODS

### Study design and sampling

The study was conducted in Halland on the western coast and Uppsala-Örebro counties in the central-eastern part of Sweden. Both regions are densely populated and have cattle herds grazing near lakes and streams. The reason for targeting these regions was that the municipalities in both regions had discussed restrictions on grazing such pastures. In total, 30 herds were recruited. According to sample size calculations, 73 herds would be needed to estimate 95% herd prevalence with 95% confidence interval (CI) assuming a perfect test. The 95% herd prevalence was based on our previous dairy herd studies (Silverlås *et al.* 2009; Silverlås & Blanco-Penedo, 2013). Because all calves up to 90 days of age were sampled and longitudinal studies show that all calves in infected herds shed oocysts at some stage during their first months (O'Handley *et al.* 1999; Santín *et al.* 2008), we assumed that a smaller herd sample size could be used. The study herds, 15 in each region, were a convenience sample of all suckler herds with more than 10 cows. Eligible herds in these regions were identified by Växa Sverige, an organisation owned by Swedish farmers, with knowledge about the cattle herds in the regions. In the middle region, eligible herds were also identified through a farmer network for local products. The owners were contacted by phone and by mail and those agreeing to participate were visited by a veterinarian once either during April–June 2012 (*n* = 24) or April–May 2013 (*n* = 6).

At the visit, rectal faecal samples were collected directly from all calves that were younger than 3 months. A brief clinical examination was done of the sampled animals. Samples were sent by mail when samplings were performed in the southern region and reached the laboratory within 3 days after sampling. Samples from the middle region were returned to the laboratory by car on the day of sampling. Information about the herds and management routines were collected from herd records and through interviews with the owners using a predetermined questionnaire. The information included e.g. herd size and number of animals in different age groups, purchase of animals, routines for calving and colostrum intake, cleaning of the calving area, mortality in neonatal calves and prevalence of diarrhoea and/or respiratory problems. Questions about age of calves when let out on pasture, if they were first kept in a temporary paddock, if the farm had pasture close to water and handling of calf manure were also included. All samples were collected before the pasture period had started.

#### Laboratory methods

Faecal consistency and colour were noted at arrival to the laboratory. One gram of each sample was cleaned and concentrated by saline-glucose flotation (Maddox-Hyttel *et al.* 2006). Ten *μ*l of each cleaned sample plus 50 *μ*l distilled water was put on teflon printed 3-well slides (Immuno-Cell, Mechelen, Belgium), dried and stained with FITC-labelled monoclonal anti-*Cryptosporidium* antibodies (CryptoCel IF test kit, Cellabs, Sydney, Australia). The entire wells were examined by epifluorescence microscopy and oocysts enumerated at 200 × magnification. The lower detection limit of this method is approximately 400 oocysts per gram faeces (OPG).

All samples that were *Cryptosporidium* positive by microscopy were further analyzed at the 18S rRNA and the 60 kDa glycoprotein (GP60) genes to determine species and *C. parvum* subtype, respectively. DNA was extracted from concentrated samples using a combined freeze-thawing and QIAamp DNA stool mini kit (QIAGEN, Hilden, Germany) protocol (Silverlås *et al.* 2010*b*). Samples were then subjected to nested polymerase chain reaction (PCR) protocols to amplify the 18S and GP60 genes. Amplification of the 18S gene was done essentially as described by Xiao *et al.* (1999, 2000). The first amplification mixture contained 1X of Platinum Taq buffer (Life Technologies), 3, 4 or 6 mm MgCl_2_, 200 *μ*m of each deoxynucleoside triphosphate, 200 *μ*m of each of primary primers, 0·16 mg/ml bovine serum albumin (BSA), 2 *μ*l DNA solution and 1 unit of Platinum Taq polymerase in a total volume of 25 *μ*l. After initial denaturation at 95 °C for 5 min, 35 cycles followed consisting of 94 °C for 45 s, 56 °C for 45 s and 72 °C for 60 s, and a final extension of 72 °C for 7 min. For the second amplification, 2 *μ*l from the first reaction were added to a reaction mixture as above except containing secondary primers and MgCl_2_ at 3 mm concentration. The reaction condition was 95 °C for 5 min followed by 40 cycles of 94 °C for 45 s, 58 °C for 90 s and 72 °C for 60 s and then a final extension step of 72 °C for 7 min. DNA samples that failed to yield a PCR product were rerun with primers designed by (Silva *et al.* 2013) following the published protocol except that bovine serum albumin (BSA), 0·16 mg/ml, was added and that 3 mm MgCl_2_ instead of 1·5 mm was used. Amplification of the GP60 gene was done according to Chalmers *et al.* (2005).

Species and subtype were determined by sequencing in both directions on an ABI 3100 Genetic Analyzer (Applied Biosystems) using the internal primers and BigDye Terminator v3·1 Cycle Sequencing Kit (Applied Biosystems). CLC Main Workbench (CLC bio, Aarhus, Denmark) was used to assemble consensus sequences and manually correct mismatches. Achieved sequences were compared with sequences deposited in GenBank using Basic Local Alignment Search Tool (BLAST, NCBI http://www.ncbi.nlm.nih.gov/blast/Blast.cgi).

#### Statistical analysis

Data were entered into Microsoft Office Excel 2007 spreadsheets (©2006 Microsoft Corporation). Statistical analyses were performed using Stata 11 (Stata Software, StataCorp LP College Station, TX, USA). Continuous normally and non-normally distributed data were analyzed using the Mann–Whitney test. Non-continuous data were categorized and analyzed using the *χ*^2^-test. A random effects logistic regression model was built, using manual forward selection and herd as random effect, to investigate factors associated with being *Cryptosporidium* positive or not. Spearman rank correlations were investigated prior to modelling to avoid collinearity between factors entered into the model. Calves were considered to be clustered within herds. Variables significant at *P* ⩽ 0·2 in univariable random effects logistic regression were considered for building the main effects model. A flow chart of these variables was done to identify any confounders or intervening factors. Confounding was considered to be present if any variable OR changed >20% when a new variable was entered into the model. The main effects model was investigated for interactions and the fit, sensitivity and specificity and distribution of residuals.

## RESULTS

The 30 herds included in the study had 10 to 99 cows (median 39) and between three and 18 calves were sampled in each herd (median 11·5). The calves were of different breeds, mainly pure bred or cross bred Angus, Charolais, Hereford, Limousine, Simmental, Holstein Friesian and Swedish Red. In total, samples were collected from 332 calves 1 to 90 days of age. One calf had poor general condition and one had tachycardia, otherwise no signs of illness were found in sampled calves.

**Fig. 1.**
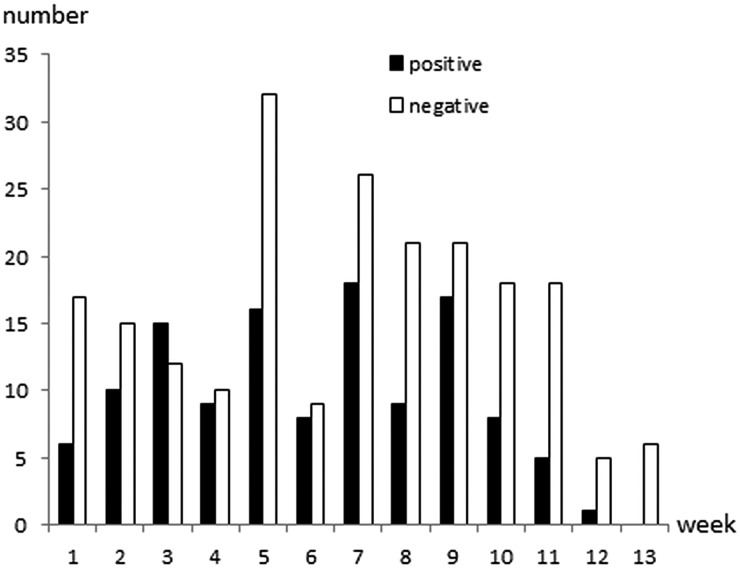
Age and *Cryptosporidium* spp. oocyst shedding of 332 calves sampled in 30 Swedish suckler herds. Faecal samples were cleaned and concentrated by saline-glucose flotation, stained with FITC-labelled monoclonal anti-*Cryptosporidium* antibodies and examined by epifluorescence microscopy. The lower detection limit of the method is approximately 400 oocysts per gram faeces (OPG; Maddox-Hyttel *et al.* 2006).

*Cryptosporidium* oocysts were detected in 122 (36·7%; 95% CI 31·5–42·1%) calves aged 1–81 days of age (Fig. 1). The faecal consistency was watery, loose, pasty and solid in 20, 79, 206 and 27 calves, respectively. Watery and loose stools were considered as diarrhoea, whereas pasty and solid were considered as normal consistency. There was no significant association between age, faecal consistency or colour of faeces, and *Cryptosporidium* infection (Table 1). Oocyst counts in the positive samples were 400 to 2·4 × 10^7^ OPG. The two youngest positive calves were only 1 and 2 days old and they shed 1600 and 400 OPG, respectively. Calves positive for *Cryptosporidium* spp. were identified in 29 (97%) herds and the within-herd prevalence varied between 6·3% and 75·0% (median 42·3%). Of the sampled calves, 267 were ⩽ 62 days, and *Cryptosporidium* oocysts were detected in 106 (39·7%) of them.

**Table 1.** Age, faecal consistency and faecal colour in 122 *Cryptosporidium* spp. positive and 210 *Cryptosporidium* spp. negative calves in 30 Swedish suckler herds

	*Cryptosporidium* spp		Test	*P*
	Positive	Negative		
Age in days				
Mean (s.d.)	39·1 (23·0)	43·5 (20·3)	Mann–Whitney test	0·096
Faecal consistency (number of calves)				
Normal	89	144	*χ*^2^-test	0·400
Diarrhoea	33	66		
Faecal colour (number of calves)				
Brown	95	172	*χ*^2^-test	0·372
Yellow	27	38		

**Fig. 2.**
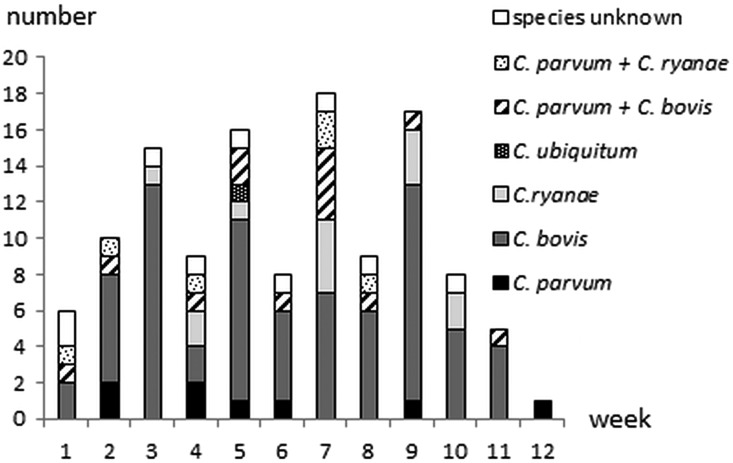
*Cryptosporidium* species distribution in 122 oocyst shedding calves in 30 Swedish suckler herds Faecal samples were cleaned and concentrated by saline-glucose flotation, stained with FITC-labelled monoclonal anti-*Cryptosporidium* antibodies and examined by epifluorescence microscopy. The positive samples were further analyzed at the 18S rRNA and the 60 kDa glycoprotein (GP60) genes to determine *Cryptosporidium* species.

*Cryptosporidium* species could be determined in 113 (92·6%) of the 122 positive samples. Monoinfection with *C. bovis* was identified in 72 (21·7%) calves, *C. ryanae* in 13 (3·9%) calves, *C. parvum* in 8 (2·4%) calves and *C. ubiquitum* in 1 (0·3%) calf. In addition, mixed *C. bovis/C. parvum* infection was identified in 13 (3·9%) calves and mixed *C. ryanae/C. parvum* infection in 6 (1·8%) calves. Thus, in total *C. parvum* was identified in 27 of the 332 faecal samples (8·1%; 95% CI 5·4–11·6%) and in 23·9% (95% CI 16·4–32·8%) of *Cryptosporidium* positive samples with successful sequence results. Nine calves shedding *C. parvum* had watery or loose stools whereas 18 had normal faecal consistency. Species distribution per week in life is shown in Fig. 2. *C. bovis, C. ryanae* and *C. parvum* were detected in 25, 13 and 9 herds, respectively. *C. bovis* infection was identified in a 1-day-old and two 4-days-old calves (one of them with mixed *C. bovis*/*C. parvum* infection). Mixed *C. ryanae/C. parvum* infection was identified in a 7-days-old calf. There was no significant age difference for the different species (*P* = 0·09–0·89, Mann–Whitney test).

Oocyst excretion ranges were 400–2·6 × 10^6^ OPG (median 9600 OPG) for *C. bovis*, 800–4·4 × 10^5^ OPG (median 4800 OPG) for *C. ryanae*, 400–3·8 × 10^6^ OPG (median 1600 OPG) for *C. parvum*, 1·3 × 10^6^ OPG for *C. ubiquitum,* 400–2·4 × 10^7^ OPG (median 8800 OPG) for mixed *C. bovis/C. parvum* and 800–2·0 × 10^7^ OPG (median 3·7 × 10^5^ OPG) for mixed *C. ryanae/C. parvum*. No significant differences in OPG were found for the different species (*P* = 0·08–0·87, Mann–Whitney test).

GP60 analysis identified two subtypes of *C. parvum*: IIaA16G1R1 in 7 calves and IIdA27G1 in 16 calves with mono or mixed *C. parvum* infection. The subtype could not be determined for the remaining four *C. parvum* positive calves. Only one subtype was detected in each herd; two herds had IIaA16G1R1, five herds had IIdA27G1 and the subtype could not be determined in two herds that had only one *C. parvum* positive calf each.

Univariable modelling returned three variables with *P* ⩽ 0·2: (1) routines for cleaning calf pens, (2) number of cows in calving pens and (3) per cent of dead (for any reason) calves the season of sampling. The flow chart identified variable (3) as intervening to both (1) and (2), i.e. the outcome of (3) could be affected by the outcome of the other variables. Another problem was that all levels of (3) were not represented for all levels of (1) and (2), causing interpretation problems. Intervening variables should not be included in modelling, and thus (3) was removed. Both variable (1) and (2) could be kept in the model, which was highly significant and fit the data well (Table 2). There was no interaction between the variables. The sensitivity and specificity of the model was low (Se 0·32, Sp 0·86), and the area under the receiver operating curve (ROC) was only 0·59. One herd was identified as an outlier by residual distribution, but was kept in the model since data were properly recorded.

**Table 2.** Variables significant at *P* ⩽ 0·2 in univariable modelling and final random effects logistic regression model of variables associated with a calf being infected with *Cryptosporidium* spp. at the time of sampling

Variable	Level	*P*_univariable_	OR_final model_	*P*_final model_	95% CI
(1) Cleaning routines for calving pens	Once a year	0·020^a^	1	0·0035^a^	
	More often		0·25	0·004	0·10–0·64
(2) Number of cows in calving pen	1 (single pen)	0·056^a^	1	0·0109^a^	
	5–15		2·5	0·029	1·10–5·89
	20–28		1·2	0·579	0·57–2·69
	30–90		0·47	0·202	0·15–1·49
	No data		5·1	0·100	0·73–35·23
(3) Per cent dead calves this year	0 1–4 5–9 ⩾10	0·054^a^		Not included in the final model	

Herd was used as random effect. Walds *χ*^2^ of final model = 20·3, 5 df, *P* = 0·0011.

a Overall *P*-value of variable

*P* = *P*-value, OR = Odds Ratio, CI = Confidence Interval

## DISCUSSION

The present study showed that *Cryptosporidium* spp. are common in Swedish suckler herds. Oocysts were detected in 29 of 30 investigated herds and in 36·7% of the sampled calves. The herd prevalence was in accordance with what has been found in Swedish dairy cattle (Silverlås *et al.* 2009; Silverlås and Blanco-Penedo, 2013). However, the prevalence among the calves was lower than in dairy calves, where 49–52% of calves have been reported to shed oocysts (*χ*^2^-test; *P* = 0·002). The study on dairy calves only included calves up to 62 days of age and accordingly only beef calves up to this age were included in the comparison. This difference is consistent with findings in other studies reporting a higher prevalence in dairy calves compared with calves in suckler herds in e.g. Belgium, Canada and Zambia (Dixon *et al.* 2011; Geurden *et al.* 2006, 2007).

The most common species was *C. bovis*, followed by *C. parvum* and *C. ryanae.* This is in concordance with the situation in our dairy herds where *C. bovis* is the most prevalent species in preweaned calves (Silverlås *et al.* 2010*b*). In Sweden, *C. parvum* has previously only been reported in cattle younger than 9 weeks, with the main window of infection before 6 weeks of age (Silverlås *et al*. 2013), but in this study we detected it in two calves, 71- and 81-days-old, respectively. This could be an effect of a lower infection pressure in suckler herds resulting in calves getting infected later. In dairy herds, calves are commonly infected before they are 3 weeks old (Santín *et al.* 2008). In suckler herds the calves are kept with their dams on a larger area, as compared with dairy calves that are kept close together, exposing them to a lower infection pressure. A common advice to prevent *Cryptosporium* infection is to ensure that all newborn calves ingest an adequate amount of colostrum during their first 24 h of life (Robertson *et al.* 2014). In Sweden, it is a legal requirement that calves are fed colostrum within 6 h after birth. Thus, there should not be a major difference in access to colostrum between beef and dairy calves.

The maximum OPG value in *C. parvum* monoinfected calves was 3·8 × 10^6^ OPG in a 12 days old calf. The other calves shed fewer oocysts and no calf older than 5 weeks shed more than 2800 OPG. The majority of calves in which *C. parvum* was detected had mixed infections where either *C. bovis* or *C. ryanae* was the only species detected by sequencing of the 18S rRNA locus. Presence of *C. parvum* was only identified when GP60 was used as an additional tool, indicating a higher infection level of the apathogenic species. Each herd was only visited once, so the maximum oocyst shedding could possibly have been higher in some individuals at other time points. Even so, together these results indicate that calves in Swedish suckler herds, where calves are usually 5–6 weeks old before turn out to pasture, are of limited importance for *C. parvum* oocysts shedding to the environment.

*C. ubiquitum* has a broad host range and wide geographic distribution and has mostly been found in sheep and humans (Fayer *et al.* 2010; Li *et al.* 2014). Cattle can be experimentally infected (Fayer *et al.* 2010), and natural infection with *C. ubiquitum* has been described once, in a 22 week old veal calf in Brittany, France (Follet *et al.* 2011). We report here the first finding of *C. ubiquitum* in cattle in Sweden. The positive sample came from a 5 week old bull calf in a herd where 11 other calves were sampled. The sequence did not contain ambiguities but was short (227 bp). PCR and sequencing of the 18S rRNA locus for this sample was thus re-run in order to confirm the result. None of the other samples from this herd contained *C. ubiquitum*.

The two *C. parvum* subtypes identified both belonged to zoonotic subtype families; IIa and IId. This is in accordance with what has been found in other European countries, where subtype family IIa is the most common in calves (Robertson *et al*. 2014). Subtype IIaA16G1R1 has previously been detected in calves and humans in Sweden (Insulander *et al.* 2013; Silverlås *et al.* 2010b, 2013). Recently this subtype of *C. parvum* caused an outbreak in Sweden through direct contact between calves and humans (Kinross *et al*. 2015). It is a common subtype in calves in Eastern Europe (Soba and Logar, 2008; Imre *et al.* 2011; Kváč *et al.* 2011) and has been reported from Germany, the Netherlands and Estonia (Broglia *et al.* 2008; Wielinga *et al.* 2008; Lassen *et al.* 2014). It has been found in pigs and lambs (Kváč *et al*. 2009; *Imre et al*. 2013), and human infection has been reported from e.g. Slovenia and Estonia (Soba and Logar, 2008; Lassen *et al.* 2014). The IIdA27G1 subtype has not been previously identified in Sweden and only two short sequences from calves, both from Romania, are present in GenBank (GB); IIdA27G1a (GB id KC469693) and IIdA27G1b (GB id KC469694). The post repetitive sequences of our isolates contained numerous double peaks in the chromatograms and it could not be determined whether they belonged to either of these subtypes or should have another small letter suffix. Subtype IIaA16G1R1 was the most common subtype found in calves from herds with calf diarrhoea problems (Silverlås *et al.* 2013). In the present study, however, none of the investigated herds reported any calf diarrhoea at the time of sampling. The distribution pattern found here, with only two subtypes in the study population and one subtype in each investigated herd, is rather uncommon and was surprising to us. A previous study from Sweden reported as many as 27 subtypes from 171 investigated *C. parvum* positive samples (Silverlås *et al.* 2013). A similar pattern with multiple subtypes has also been observed in other molecular epidemiology studies performed in Europe, although the subtype diversity varies greatly among countries (reviewed by Robertson *et al*. 2014). The highest GP60 diversity, besides the Swedish study, has been reported from the Netherlands, where 17 subtypes were found in 100 investigated *C. parvum* positive samples (Wielinga *et al*. 2008). It has been suggested that herd management strategies affect subtype distribution. Areas with closed herd management (few animal movements between herds) usually show a high number of subtypes in the calf population, but only one subtype in each herd (Misic & Abe, 2007; Brook *et al.* 2009; Soba & Logar, 2008; Silverlås *et al.* 2013). In contrast, fewer subtypes but two or more subtypes per herd is a common pattern in areas with animal exchange between herds (Peng *et al.* 2003; Trotz-Williams *et al.* 2006; Brook *et al.* 2009). The vast majority of the herds in the present study had bought animals during recent years so we expected to find the latter pattern here.

The median herd size in this study was larger than the overall median suckler herd size in Sweden at the time of the study. It is thus possible that our data is biased compared with the average *Cryptosporidium* spp. infection pressure and species distribution in Swedish suckler herds. Infection pressure generally increases with population size, which could mean that we have overestimated both the herd and the within-herd prevalence of *Cryptosporidium* spp. It is possible more herds would have come out as *Cryptosporidium* spp. negative if smaller herds would have been included to a larger extent. Another possible bias is that the small sample size (30 herds) may not be representative of all suckler herds. Increasing the sample size certainly increases the reliability of results but this could not be done due to lack of funding. The model identified two factors associated with *Cryptosporidium* infection. However, the sensitivity, specificity and prediction probability of the model was low, indicating that other important factors were missed. The reasons could be that the questionnaire was largely developed based on knowledge about routines in dairy herds, and also that even though questions were categorized farmers were allowed to give more open answers instead of just checking the most applicable answer. This led to a number of answers spanning several categories, or making it impossible to categorize answers and a number of variables could not be used for the regression analysis. There was no indication of unstable estimates due to few observations. The reason for one herd becoming an outlier in the model is most likely that this was the only herd that had not answered the question about the number of cows in calving pens, creating a unique level of that variable for those calves.

In conclusion, the results show that *Cryptosporidium* infection is common in Swedish suckler herds and that *C. bovis* is the dominant species in young calves. Approximately 8% of the calves excreted *C. parvum* which is consistent with earlier studies in the dairy herds. The two subtypes of *C. parvum* that were found both belong to zoonotic subtype families.
